# Extracorporeal membrane oxygenation (ECMO) in patients with severe COVID-19 adult respiratory distress syndrome: a systematic review and meta-analysis

**DOI:** 10.1186/s43057-021-00046-3

**Published:** 2021-04-12

**Authors:** Hany Hasan Elsayed, Aly Sherif Hassaballa, Taha Aly Ahmed, Mohammed Gumaa, Hazem Youssef Sharkawy

**Affiliations:** 1 Thoracic Surgery Department, Ain Shams University, Abbasia Square, Cairo, Egypt; 2 Cardiothoracic Surgery Department, Ain Shams University, Cairo, Egypt; 3 TRUST Research centre, Cairo, Egypt

**Keywords:** ECMO, COVID-19, ARDS, Venovenous

## Abstract

**Background:**

COVID 19 is the most recent cause of adult respiratory distress syndrome (ARDS). Extracorporeal membrane oxygenation (ECMO) can support gas exchange in patients failing conventional mechanical ventilation, but its role is still controversial. We conducted a systematic review and meta-analysis on ECMO for COVID-associated ARDS to study its outcome.

**Main body:**

CENTRAL, MEDLINE/PubMed, Cochrane Library, and Scopus were systematically searched from inception to May 28, 2020. Studies reporting five or more patients with COVID-19 infection treated venovenous with ECMO were included. The main outcome assessed was mortality and ICU/hospital discharge. Baseline, procedural, outcome, and validity data were systematically appraised and pooled with random-effect methods. The validity of all the included observational studies was appraised with the Newcastle Ottawa scale. Meta-regression and publication bias were tested. This trial was registered with PROSPERO under registration number CRD42020183861.

From 1647 initial citations, 34 full-text articles were analyzed and 12 studies were selected, including 194 patients with confirmed COVID-19 infection requiring ICU admission and venovenous ECMO treatment. Median Newcastle-Ottawa scale was 6 indicating acceptable study validity. One hundred thirty-six patients reached an endpoint of weaning from ECMO with ICU/hospital discharge or death while the rest were still on ECMO or in the ICU. The median Berlin score for ARDS prior to starting ECMO was III. Patients received mechanical ventilation before ECMO implementation for a median of 4 days and ECMO was maintained for a median of 13 days. In hospital and short-term mortality were highly variable among the included studies ranging between 0 and 100%. Random-effect pooled estimates suggested an overall in-hospital mortality risk ratio of 0.49 (95% confidence interval 0.259 to 0.721; *I*^2^ = 94%). Subgroup analysis according to country of origin showed persistent heterogeneity only in the 7 Chinese studies with pooled estimate mortality risk ratio of 0.66 (*I*^2^ = 87%) (95% CI = 0.39-0.93), while the later larger studies coming from the USA showed pooled estimate mortality risk ratio of 0.41 (95% CI 0.28-0.53) with homogeneity (*p*=0.67) similar to France with a pooled mortality risk ratio of 0.26 (95% CI 0.08-0.43) with homogeneity (*p*=0.86). Meta-regression showed only younger age as a predictor of mortality (*p*=0.02). Publication bias was excluded by visualizing the funnel plot of standard error, Egger’s test with *p*=0.566, and Begg and Mazumdar test with *p*=0.373.

**Conclusion:**

The study included the largest number of patients with outcome findings of ECMO in this current pandemic. Our findings showed that the use of venovenous ECMO at high-volume ECMO centers may be beneficial for selected COVID 19 patients with severe ARDS. However, none of the included studies involve prospective randomized analyses; and therefore, all the included studies were of low or moderate quality according to the Newcastle-Ottawa scale. In the current era and environment of the pandemic, it will likely be very challenging to conduct a prospective randomized trial of ECMO versus no-ECMO for COVID-19. Therefore, the information contained in this systematic review of the literature is valuable and provides important guidance.

**Trial registration:**

The study protocol link is at www.crd.yorl.ac.uk/PROSPERO under registration number CRD42020183861.

## Background

Coronavirus disease 2019 (COVID-19) is a viral respiratory tract infection caused by a coronavirus which was first documented in Wuhan, China, in December 2019 [1].

After then, this outbreak spread globally and has been considered as a pandemic and an international public health emergency by the WHO since March 11, 2020. As of 6th of September 2020, a cumulative total of 27,083,427 confirmed cases of coronavirus disease 2019 (COVID-19) were reported with total 884,029 deaths in 203 countries and territories worldwide [2]. Currently, there is neither a proven effective medication nor a vaccine has been discovered for the COVID-19 infection.

Although most patients with COVID-19 infection have only mild or uncomplicated course, around 10-20% will develop a severe disease that necessitates hospitalization and oxygen therapy or even ICU admission and progression to acute respiratory distress syndrome (ARDS). The prevalence of ARDS caused by COVID-19 is around 8.2% who will require mechanical ventilation and prone positioning [1]. Furthermore, hyperinflammatory state by cytokine storm appears to be a solid part of severe COVID-19 disease [3].

However, a group of patients will suffer from persistent hypoxemia and intractable ARDS despite maximum conventional treatment with mechanical ventilation and mortality among this subgroup is markedly high. Initial reports from China, Italy, and the USA suggest high patient admission to intensive care units (ICU) and mechanical ventilation with shockingly very high mortality rate among patients with severe ARDS due to COVID 19 [4–8].

Another option for severe refractory ARDS patients is venovenous extracorporeal membrane oxygenation (ECMO) which is considered as a rescue therapy. ECMO appeared to be beneficial during the influenza A (H1N1) pandemic back in 2009, with a mortality rate of 21% [9]. In another observational study on patients with H1N1-related ARDS, the mortality rate was 23.7% for ECMO patients in versus 52.5% for non ECMO patients [10]. In 2018, a retrospective study was conducted on Middle East respiratory syndrome (MERS) patients with refractory ARDS and showed that ECMO should be used as a rescue therapy because it is associated with lower mortality when compared to conventional mechanical ventilation group (65 vs. 100%, *p* = 0.02) [11]. No ECMO report was published on SARS (coronavirus emerging in 2002)-related ARDS.

The role of ECMO in the management of COVID-19 ARDS remains unclear. The initial reports of using venovenous ECMO with COVID-19 patients suffering from intractable hypoxemia observed a high mortality rate and recommended using ECMO with caution in the current pandemic [12]. According to the interim guidance made by the World Health Organization (WHO), venovenous ECMO could be considered as a salvage therapy for COVID-19 with refractory hypoxemia in expert centers with enough cases to ensure clinical expertise [13].

In view of the current growing pandemic and the fact that only a little experience with using ECMO to support COVID-19 patients is available, we aimed to estimate the effect of venovenous ECMO on mortality from COVID-19 patients with respiratory failure via all available studies by performing a systematic review and meta-analysis.

## Main text

### Search strategy and selection criteria

Our methodology followed the reporting guidelines of Meta-analysis Of Observational Studies in Epidemiology (MOOSE) and Preferred Reporting Items for Systematic Reviews and Meta-Analyses (PRISMA) guidelines. We electronically ran a search on CENTRAL, MEDLINE/PubMed, Cochrane Library, and Scopus. On Pubmed, the word search used was (COVID OR SARS COV2 OR pandemic) AND (ARDS) OR (acute respiratory distress syndrome) OR (acute lung injury) OR (respiratory failure) OR (respiratory insufficiency) OR (ECMO) OR (extracorporeal membrane oxygenation).

We contacted authors for some missing data, searched trial registries, included the gray literature, and used studies accepted and ahead of print. We did our search from inception up to May 28, 2020, which was the date of our final search without language restrictions. We used both subject headings and text word terms to search for articles about ECMO or mechanical ventilation with ARDS in COVID-19 patients. There were no language restrictions. Inclusion criteria were (all criteria should be concomitantly met for study inclusion) (a) study reporting on 5 or more patients with final outcomes; (b) with confirmed COVID 19 infection; (c) receiving venovenous ECMO. Exclusion criteria were (one criterion was sufficient for study exclusion) (a) inclusion of <5 patients with COVID-19 infection treated ECMO (thereby, any study reporting on fewer than 5 patients or case reports treated with ECMO were excluded); (b) duplicate publication (in which case only the most recent report from the same study group was included in the systematic review). Use of a sample size cut-off was chosen to limit the risk of imprecision and publication bias. (c) Studies in which the main focus was veno-arterial ECMO for treating COVID-19 or (d) studies with insufficient data about outcome endpoints (mortality, extubation, weaning, and discharge). AH, TA, and HY independently reviewed the titles and abstracts of all citations. Then, they independently reviewed the full text of both definite and potentially eligible studies for inclusion. Disagreements were reviewed by a fourth reviewer HE, who had a deciding vote.

### Quality assessment

The quality of the included studies was assessed using the Newcastle-Ottawa scale (NOS) [14]. This assessment tool is recommended by the Cochrane collaboration to assess risk of bias of non-randomized observational studies. The tool uses three domains: selection of study groups (four points); comparability of groups (two points); and ascertainment of exposure and outcomes (three points). Thresholds for converting the Newcastle-Ottawa scales to AHRQ standards (good, fair, and poor):

- Good quality: three or four stars in selection domain + one or two stars in comparability domain + two or three stars in outcome/exposure domain
- Fair quality: two stars in selection domain + one or two stars in comparability domain + two or three stars in outcome/exposure domain.
- Poor quality: zero or one star in selection domain OR zero stars in comparability domain OR zero or 1 stars in outcome/exposure domain.

### Data analysis

A meta-analysis was conducted to examine the mortality incidence in venovenous ECMO treatment for COVID-19. Data were summarized using the risk ratio (95% confidence interval (CI)). The data were pooled using DerSimonian-Laird random effects model [15]. *P* value of 0.05 or less was statistically significant. Cochran *Q* and *I*^2^ were used to assess heterogeneity between studies. The degree of heterogeneity was categorized as either low (*I*^2^ < 25%), moderate (*I*^2^ = 25–75%), or high (*I*^2^ > 75%) [16]. A *P* value of ≤ 0.05 indicated significant heterogeneity. A subgroup meta-analysis according to the study’s country of origin was conducted to investigate the high heterogeneity detected. Exploratory meta-regression analysis was performed to identify significant moderators using the inverse-variance weighted-least-squares linear regression analysis. Studies that included a control group with patients treated with mechanical ventilation were independently studied and mortality estimates were pooled using odds ratio and 95% CI. Secondary outcome measures included age, ventilation days before ECMO, and duration time on ECMO. Publication bias was examined by visual inspection of the funnel plot and tested by Egger’s test and Begg and Mazumdar test. A *p* value of ≤ 0.05 indicated the existence of publication bias. All analyses were performed using Open Meta Analyst software Windows 10 version.

The data used in the meta-analysis in each study were the number of mortality events and the number of closed cases (either cured or dead). Patients who were still on treatment were not included in the final analysis of cases of the study. The corresponding authors of the studies were contacted by email to provide additional information regarding the patients who were still receiving treatment. The numbers used in Jacobs et al. (2020) and Beyls et al. (2020) were not those reported by the study but rather provided by the author (unpublished data).

## Results

**Fig. 1:**
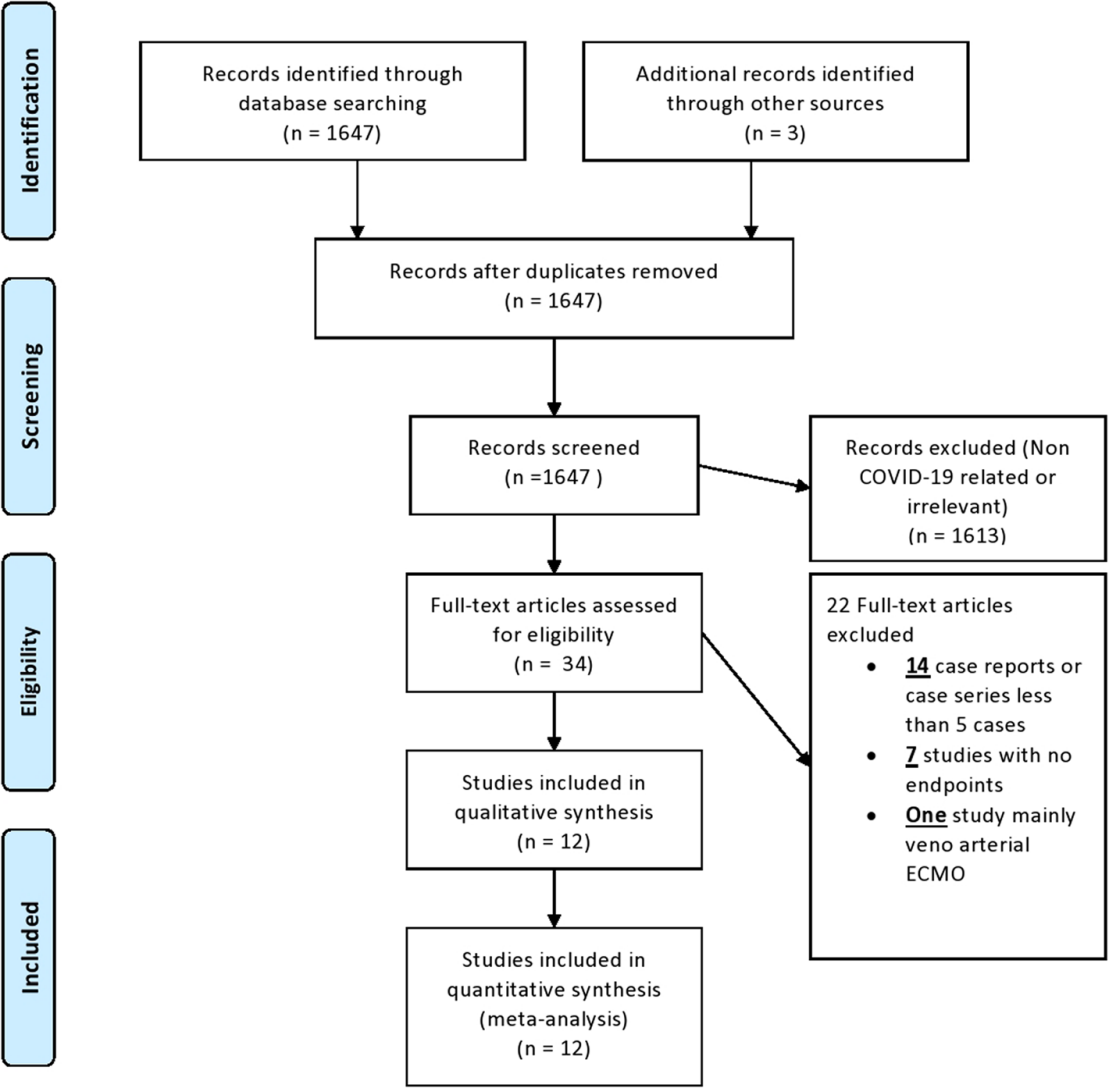
Flow diagram of study selection

Our electronic search retrieved 1647 citations, 34 of which were selected for full-text review (Fig. 1). Twelve studies with a combined population of 194 patients fulfilled the inclusion criteria (Table 1). One hundred thirty-six patients reached an endpoint of weaning from ECMO with ICU/hospital discharge or death while the rest were still on ECMO or in the ICU. Overall study validity was acceptable, with a median score of 6 on the Newcastle Ottawa scale NOS appraising the quality of observational studies, without being opposed by their non-randomized design. The median Berlin score for ARDS prior to starting ECMO was III. Patients received mechanical ventilation before ECMO implementation for a median of 4 days and ECMO was maintained for a median of 13 days (Tables 2 and 3).

**Table 1:** Characteristics and quality of the included studies

Study (author and year)	Country	Design	Setting	NOS	Follow-up	Primary end point
Zhou et al. (2020) [17]	China	Observational	Single center	4 (2,0,2)	In hospital	Death
Zhang et al. (2020) [18]	China	Observational	Single center	4 (2,0,2)	In hospital	Death
Takeda et al. (2020) [19]	Japan	Observational	Multicenter	4 (2,0,2)	In hospital	Death
Jacobs et al. (2020) [20]	USA	Observational	Multicenter	4 (2,0,2)	In hospital	Death
Yang et al. (2020) [21]	China	Observational	Single center	6 (2,2,2)	In hospital	Death
Ruan et al. (2020) [22]	China	Observational	Single center	6 (2,2,2)	In hospital	Death
Li et al. (2020) [23]	China	Observational	Single center	6 (2,2,2)	In hospital	Death
Guan et al. (2020) [24]	China	Observational	Single center	6 (2,2,2)	In hospital	Death
Zeng et al. (2020) [25]	China	Observational	Single center	4 (2,0,2)	In hospital	Death
Beyls et al. (2020) [26]	France	Observational	Single center	5 (3,0,2)	4 weeks after discharge	Death
Osho et al. (2020) [27]	USA	Observational	Single center	4 (2,0,2)	In hospital	Death
Haye et al. (2020) [28]	France	Observational	Single center	4 (2,0,2)	In hospital	Death

*NOS* Newcastle-Ottawa scale (total score with 3 numbers of subdivision: selection of study groups, comparability of groups, and ascertainment of exposure)

**Table 2:** Pre ECMO patient characteristics

Study (author and year)	Patients admitted to ICU	Patients receiving ECMO (total =194)	Age	ARDS Berlin grading	Days of ventilation pre ECMO
Zhou et al. (2020) [17]	59	5	55.6	N/A	5 (1-20)
Zhang et al. (2020) [18]	48	10	55	GRADE II	N/A
Takeda et al. (2020) [19]	N/A	26	71	GRADE III	3 (0-9)
Jacobs et al. (2020) [20]	N/A	85	52	GRADE III	4 (2.5-6.5)
Yang et al. (2020) [21]	N/A	6	59.7	N/A	N/A
Ruan et al. (2020) [22]	62	7	50	N/A	N/A
Li et al. (2020) [23]	20	8	64.25	GRADE III	6 (0-21)
Guan et al. (2020) [24]	37	5	47	N/A	N/A
Zeng et al (2020) [25]	12	12	50.9	N/A	N/A
Beyls et al. (2020) [26]	N/A	16	62	N/A	4 (1.5-7.5)
Osho et al. (2020) [27]	N/A	6	47	N/A	5.5 (3.5-6.75)
Haye et al. (2020) [28]	N/A	8	57.2	GRADE III	4 (1-9)

*N/A* not available

**Table 3:** Main outcome of ECMO patients

Study (author and year)	Veno venous ECMO	Duration on ECMO (days)	Patient endpoint (weaned/death) (total=136)	Mortality from ECMO
Zhou et al. (2020) [17]	100%	10 (5-16)	5	0 (0%)
Zhang et al. (2020) [18]	100%	N/A	5	3 (60%)
Takeda et al. (2020) [19]	100%	N/A	16	0 (0%)
Jacobs et al. (2020) [20]	100%	N/A	50	21 (42%)
Yang et al. (2020) [21]	100%	N/A	5	5 (100%)
Ruan et al. (2020) [22]	100%	N/A	7	7 (100%)
Li et al. (2020) [23]	100%	37 (9-47)	6	3 (50%)
Guan et al. (2020) [24]	100%	N/A	5	5 (100%)
Zeng et al. (2020) [25]	100%	11 (3-28)	8	5 (63%)
Beyls et al. (2020) [26]	100%	16 (2-28)	16	4 (25%)
Osho et al. (2020) [27]	100%	12 (4-18)	6	2 (33%)
Haye et al. (2020) [28]	100%	14 (8-28)	7	2 (29%)

**Fig. 2:**
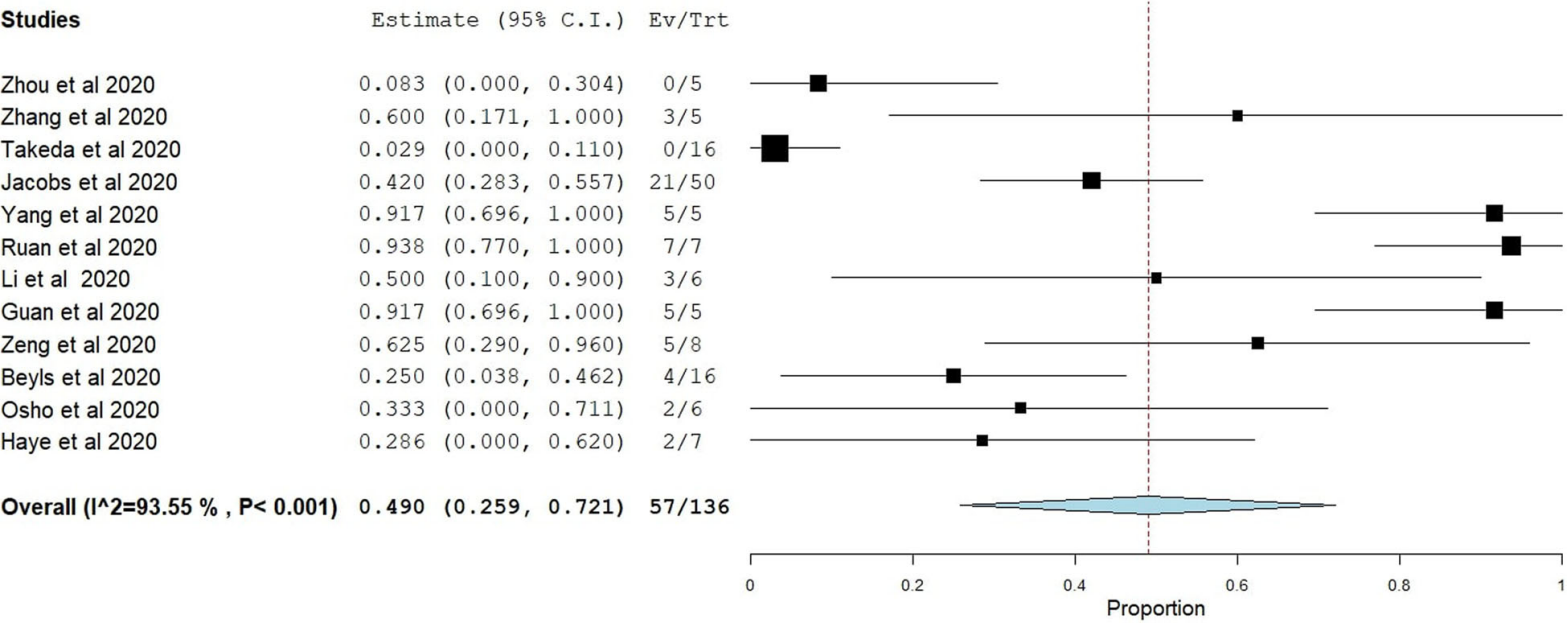
Forest plot of pooled analysis of mortality by random effect model in all studies of ECMO with COVID-19 patients

Random-effect pooled estimates suggested an overall in-hospital mortality risk ratio of 0.49 (95% confidence interval 0.259 to 0.721; *I*^2^ = 94%) (Fig. 2). Most of the preliminary studies were from China (seven studies with 41 patients having endpoints). Larger studies then followed from the USA, Japan, and France (Five studies with 95 patients with endpoints).

**Fig. 3:**
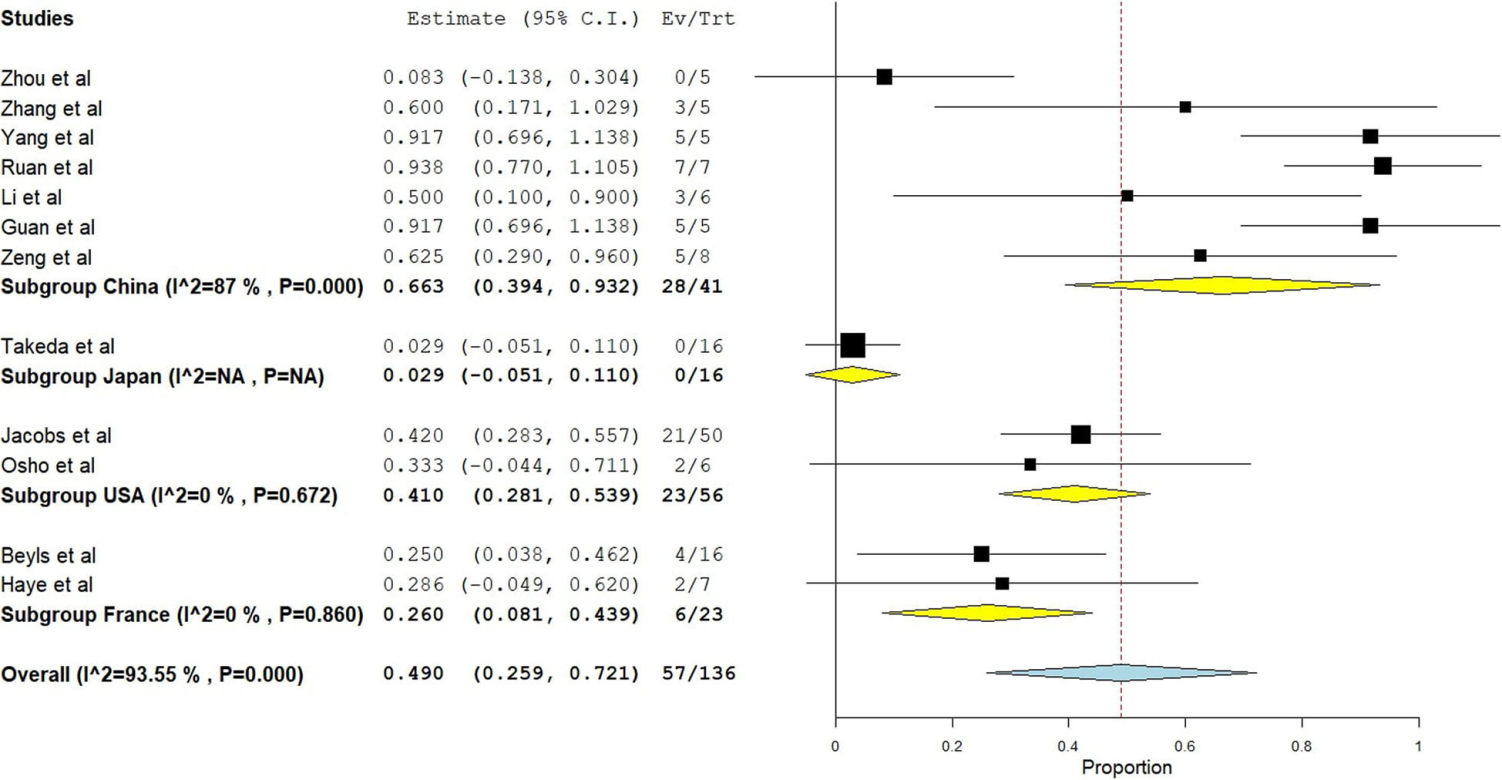
Forest plot of pooled analysis of mortality by random effect model in all studies of ECMO with COVID-19 patients with subgroup division of country of origin

To investigate the overall inter-study heterogeneity, a subgroup analysis was performed according to the country of origin of each study (Fig. 3). This showed persistent heterogeneity only in the 7 Chinese studies with pooled mortality risk ratio of 0.66 (*I*^2^ = 87%) (95% CI = 0.39-0.93), while the later larger studies coming from the USA showed pooled estimate mortality risk ratio of 0.41 (95% CI 0.28-0.53) with homogeneity (*p*=0.67) similar to France with a pooled mortality risk ratio of 0.26 (95% CI 0.08-0.43) with homogeneity (*p*=0.86).

**Fig. 4:**
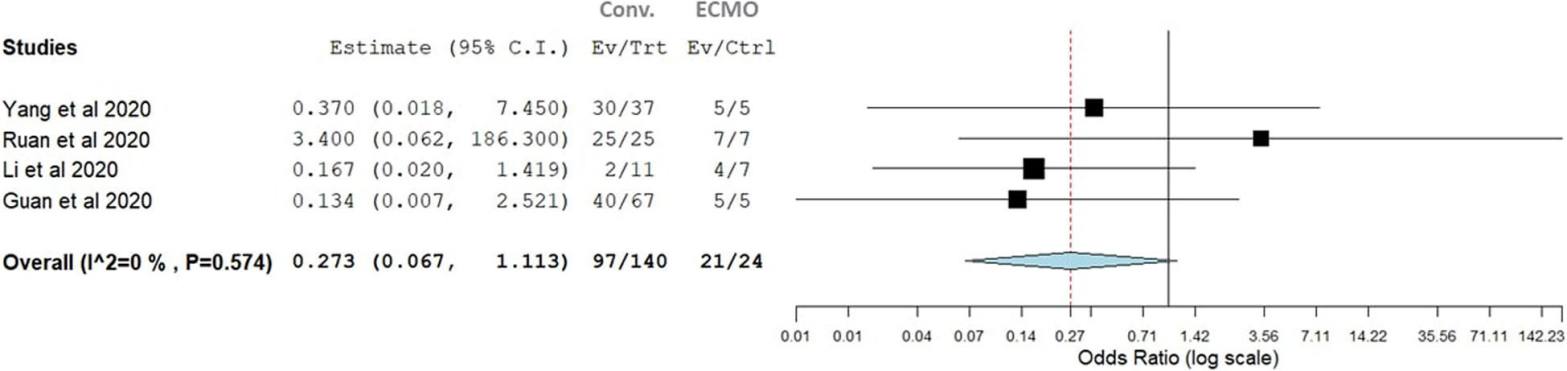
Forest plots for odds of mortality in COVID-19 patients receiving ECMO therapy versus conventional invasive mechanical ventilation therapy

In four of our studies, there was a control group who received mechanical ventilation for severe ARDS. The mortality rate was 87.5% in the ECMO patients and 69.2% in conventional therapy patients. The pooled odds of mortality in ECMO versus conventional therapy were not significantly different (*p*=0.273, 95% CI, 0.06–1.111). There was no observable heterogeneity (*I*^2^ = 0%, Cochran’s *Q*, *p* value = 0.57 ((Fig. 4).

**Fig. 5:**
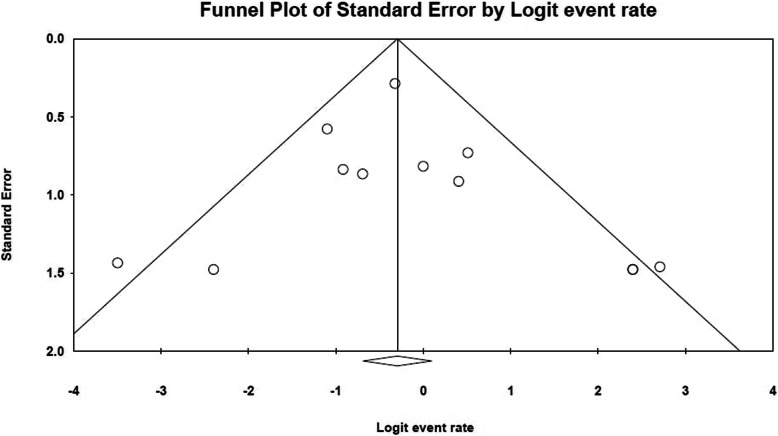
Funnel plot of standard error

Moderators tested were age, Pre ECMO-Berlin score of ARDS, ventilation days before ECMO, and duration of days on ECMO. Exploratory meta-regression identified age as a significant negative moderator of mortality (*p* =0.02) with younger age patients at a higher risk of death. No other factors demonstrated a significant moderator but this lack of a statistical significance for likely or established prognostic factors should be viewed with consideration of the limited statistical power of meta-regression when applied to a limited dataset. Publication bias was excluded by visualizing the funnel plot of standard error (Fig. 5). The funnel plot is symmetrical with only 2 studies outside the threshold [19, 22]. Exclusion of bias was also proven with an Egger’s test value *p*=0.566 and Begg and Mazumdar test value with *p*=0.373.

## Discussion

To our knowledge, this is the first systematic review and meta-analysis of the use of venovenous ECMO in treating severe COVID-19-related ARDS. We identified 12 independent studies [17–28] that provided data on the outcome of ECMO in COVID-19 patients. The initial studies testing the efficacy of ECMO in COVID-19 were small and pessimistic. A study by Ñamendys-Silva SA [29] looked at pooled mortality and efficacy from initial Chinese reports (only 17 patients). It included 2 of our included studies [21, 24] and found a mortality of 82.3% (14/17) with no overall benefit of ECMO (*z*=0.57, *p*=0.56). Henry and Lippi [30] shortly followed by another pooled analysis which included also 2 of our included studies [21, 22] comparing ECMO (total of 17 patients) and conventional mechanical ventilation and again found no benefit for ECMO in severe COVID-19 with pooled odds of mortality in the ECMO group versus the conventional therapy group not significantly different (OR: 2.00, 95% CI: 0.49–8.16) with no observable heterogeneity (*I*^2^ = 0%, Cochran’s *Q*, *p* value = 0.99).

We performed a subgroup single arm meta-analysis for studies including another arm for invasive mechanical ventilation for severe ARDS due to COVID 19, but all our ECMO studies had 5 or more patients [21–24] as per inclusion criteria and again found no benefit or harm for ECMO in treating severe ARDS in COVID-19. The pooled odds of mortality in the ECMO group versus the conventional therapy group were not significantly different (*p*=0.273, 95% CI: 0.06–1.111). The mortality was high in both groups (87.5% vs 69.2%). We believe there are two limitations here; firstly, none of the larger more recent studies with better outcomes [19, 20, 25–28] had a control arm of treating COVID-related ARDS with conventional mechanical ventilation. Secondly, we believe these are two different populations with patients having variable levels of ARDS severity, with those receiving ECMO treatment being potentially more critically ill in some cases, which could have impacted the outcome for mortality rates.

We have shown by our subgroup analysis that heterogeneity was mainly in the initial Chinese (*I*^2^=87%) studies and the following studies showed homogeneity from the USA (*I*^2^=0%, *p*=0.67) and France (*I*^2^=0%, *p*=0.86) with lower mortality associated with the use of ECMO for severe COVID-19 patients, although the number of studies was small. The three largest case series [19, 20, 26] included in this systematic review were all outside China and showed more promising results than the rest of studies with smaller numbers [17, 18, 21–25, 27, 28]*.* A mortality of 29.2% only in these series as compared to a mortality of 61.1% in the rest of the nine studies (seven Chinese series). This may reflect the fact that high flow ECMO centers achieve superior results. Our data thus confirm these results showing the beneficial effects of ECMO in large tertiary referral centers for the treatment of COVID-19 ARDS. This is consistent with previous findings of Barbaro and his colleagues [31] who found lower ECMO case-mix adjusted mortality in adult patients (adjusted OR, 0.61; 95% CI, 0.48–0.79) in higher volume ECMO centers. Our definition of high volume centers is like them as performing more than 30 cases/year.

The role of ECMO in improving outcomes in severely ill COVID-19 patients seems to be multi factorial. The ARDS observed in COVID-19 patients mostly fits the Berlin criteria [32] but Gattinoni and his colleagues [33] have proposed that the classic ARDS injury is only present in 20-30% of COVID-19 patients with decreased pulmonary compliance less than 40 ml/cmH2O while the non-ARDS type (present in 70-80%), the severe hypoxemia is associated with a respiratory system compliance of more than 50 ml/cmH2O. Hence, severe hypoxemia is primarily due to ventilation/perfusion (VA/Q) mismatch. Unlike classic ARDS, high PEEP pressures and prone positioning in this subgroup of COVID-19 patients do not improve oxygenation through the classic theory of the recruitment of collapsed areas. Although our meta regression showed that pre ECMO days of ventilation did not affect outcome, these patients could benefit from early ECMO to avoid ventilator-induced lung injury and this was the recommendation from the only two included studies in our analysis which had zero mortality in their ECMO patients [17, 19].

Secondly, there is an increasing trend to show that COVID-19 infection is associated with a hypercoagulable and thrombotic state. Yin et al. [34] studied the differences of coagulation features between severe pneumonia caused by the SARS-CoV-2 (COVID-19) and non-SARS-CoV-2 viruses and found that platelet count of the COVID-19 group was significantly higher than that of non-COVID-19 patients. Beyls and his colleagues [26] suggested that venous-Doppler ultrasonography of femoral and jugular veins should be performed routinely for severe COVID-19-related ARDS in preparation in case ECMO therapy is needed as they found a higher rate of ECMO-related line thrombosis. ECMO circuits eliminate coagulation factors binding them irreversibly to their surface coating material. Systemic anticoagulation is usually utilized for ECMO and further aggravates the anti-coagulatory state on many levels [35]. In their current COVID-19 guidelines, the “Extracorporeal life support organisation ELSO” recommended following existing anti-coagulation guidelines, with consideration given to an anti-coagulation targeted at the higher end of normal with vvECMO given the known hyper-coagulable status of COVID-19 patients [36].

In their meta-analysis, Munshi and his colleagues [37] have shown a reduction in 60-day mortality in patients receiving ECMO for ARDS in comparison to conventional mechanical ventilation, but with an associated increased risk of bleeding. The improvement in ARDS outcome and the anti-thrombotic benefit shown in this meta-analysis is a hypothesis of the additional benefit in severely ill COVID-19 patients.

Two of our studies [20, 22] suggested that higher mortality related to patients receiving ECMO or conventional mechanical ventilation for severe ARDS can be related to cytokine production. There is accumulating evidence suggesting that a sub-group of patients with severe COVID-19 disease have a cytokine storm syndrome in which a cascade of activated cytokines leads to harmful auto-amplification of inflammatory cytokine production leading to end-organ damage and a higher risk of mortality. Among COVID-19 patients who have received ECMO, a strong positive correlation exists between mortality and high cytokine levels, most notably IL-6 [38].

Ruan et al. [22] found that Interleukin-6 concentrations differed significantly between non survivors and survivors in their COVID-19 cohort, with non survivors having up to 1.7 times higher values. We could not study this in our meta-analysis as both studies did not mention absolute values for cytokines for the ECMO only group.

Our meta-analysis suggests that there is a potential role for ECMO in appropriately selected patients with severe COVID-19. Although the risk factors and variables that contribute to the optimal outcome are complex and reflect individual ECMO center experiences and available resources during the pandemic, it can be argued that it would be unethical to withhold ECMO (or consideration for referral to an experienced ECMO center) in patients who might potentially benefit from this therapy as previously suggested by Abrams and colleagues [39] when considering ECMO for ARDS due to all causes.

The planning and execution of a randomized controlled trial of this advanced intervention during the current pandemic is difficult. Current challenges include randomization of markedly sick patients early with a higher risk of death, the need for engagement of many centers worldwide, the lack of the ECMO service in poorer and third world countries, and the inconsistency of managing the control non ECMO group. As a result, a current study of ECMO in patients with COVID-19-related severe ARDS soon is unlikely. Thus, our meta-analysis can provide clinicians with the most comprehensive synthesis of all the available limited evidence for the outcome of vvECMO in adult patients with severe ARDS due to COVID-19, although further data collection and meta-analysis for larger studies are invited.

### Limitations

The project has obvious limitations, including those which are typical of any systematic review and meta-analyses. By pooling observational studies, this review cannot overcome the limitations of its primary studies included which were relatively of small numbers (apart from one [20]) and, still none were based on a randomized allocation. Indeed, the authors believe only meta-analyses of homogeneous well-powered randomized trials should be considered a solid scientific proof of the safety and efficacy of any medical/surgical intervention which is difficult to achieve on the short-term period while we are still facing an unresolved pandemic and literature guidance from the available data is needed to support decision on a stretched medical resources setting in most countries The secondary outcome data was missing in numerous studies and the focus by the authors was mortality outcome and hence authors needed to be contacted by emails to fill in missing information. However, systematic reviews and meta-analyses of non-randomized studies (as in the current case) can be meaningful and guide current practice, even if only by emphasizing the limitations of the available clinical evidence (as in the current ECMO use with the COVID 19 pandemic). Another limitation of the outcome was the possibility of weaning from ECMO followed by ICU discharge then dying in-hospital which was not recorded at the time of study.

Furthermore, the exclusion of 14 reports because including less than 5 cases or being case reports is a call for more collaborative research efforts. This type of collaboration is essential for the present clinical challenges of the COVID-19 crisis. To complement this collaboration, ASAIO has developed a database specific to ECMO use in severe COVID-19 to aid in this effort. Merging and synergizing data between databases such as those obtained by ASAIO, ESLO, and “SpecialtyCare” may provide insight about the relevant exposure, demographics, comorbidities, and clinical and laboratory variables that may predict outcome, aid selection of patients, or even suggest futility (against the evidence presented here).

## Conclusions

The study included the largest number of patients with outcome findings of ECMO in this current pandemic. Our findings showed that the use of venovenous ECMO at high-volume ECMO centers may be beneficial for selected COVID-19 patients with severe ARDS. However, none of the included studies involve prospective randomized analyses; and therefore, all the included studies were of low or moderate quality according to the Newcastle-Ottawa scale. In the current era and environment of the pandemic, it will likely be very challenging to conduct a prospective randomized trial of ECMO versus no-ECMO for COIVID-19. Therefore, the information contained in this systematic review of the literature is valuable and provides important guidance.

## Data Availability

All data and materials are available on request.

## Abbreviations

ARDS: Adult respiratory distress syndrome
ECMO: Extracorporeal membrane oxygenation
ELSO: Extracorporeal life support organization
MERS: Middle East respiratory syndrome
UFH: Unfractionated heparin
vvECMO: Venovenous extracorporeal membrane oxygenation
ASAIO: American Society for Artificial Internal Organs

## References

[1] (2020) The epidemiological characteristics of an outbreak of 2019 novel coronavirus diseases (COVID-19) in China. Zhonghua Liu Xing Bing Xue Za Zhi 41(2):145–151. 10.3760/cma.j.issn.0254-6450.2020.02.003 10.3760/cma.j.issn.0254-6450.2020.02.003 32064853

[2] WHO (2019) Coronavirus disease (COVID-19) situation report – 107. World Heal Organ 2020:2633

[3] Zhou F, Yu T, Du R et al (2020) Clinical course and risk factors for mortality of adult inpatients with COVID-19 in Wuhan, China: a retrospective cohort study. Lancet 395(10229):1054–1062. 10.1016/S0140-6736(20)30566-3 32171076 10.1016/S0140-6736(20)30566-3 PMC7270627

[4] Grasselli G, Zangrillo A, Zanella A et al (2020) Baseline characteristics and outcomes of 1591 patients infected with SARS-CoV-2 admitted to ICUs of the Lombardy region, Italy. JAMA. 10.1001/jama.2020.5394 10.1001/jama.2020.5394 PMC7136855 32250385

[5] Richardson S, Hirsch JS, Narasimhan M et al (2020) Presenting characteristics, comorbidities, and outcomes among 5700 patients hospitalized with COVID-19 in the New York City area. JAMA. 10.1001/jama.2020.6775 10.1001/jama.2020.6775 PMC7177629 32320003

[6] Yan Y, Yang Y, Wang F, Ren H, Zhang S, Shi X, Yu X, Dong K (2020) Clinical characteristics and outcomes of patients with severe covid-19 with diabetes. BMJ Open Diabetes Res Care 8(1):e001343. 10.1136/bmjdrc-2020-001343 32345579 10.1136/bmjdrc-2020-001343 PMC7222577

[7] Yu Y, Xu D, Fu S et al (2020) Patients with COVID-19 in 19 ICUs in Wuhan, China: a cross-sectional study. Crit Care 24:219 32410714 10.1186/s13054-020-02939-x PMC7223395

[8] Chen T, Wu D, Chen H, Yan W, Yang D, Chen G, Ma K, Xu D, Yu H, Wang H, Wang T, Guo W, Chen J, Ding C, Zhang X, Huang J, Han M, Li S, Luo X, Zhao J, Ning Q (2020) Clinical characteristics of 113 deceased patients with coronavirus disease 2019: retrospective study. BMJ 368. 10.1136/bmj.m1091 10.1136/bmj.m1091 PMC7190011 32217556

[9] Australia and New Zealand Extracorporeal Membrane Oxygenation (ANZ ECMO) Influenza Investigators, Davies A, Jones D, Bailey M, Beca J, Bellomo R, Blackwell N, Forrest P, Gattas D, Granger E, Herkes R, Jackson A, McGuinness S, Nair P, Pellegrino V, Pettilä V, Plunkett B, Pye R, Torzillo P, Webb S, Wilson M, Ziegenfuss M. Extracorporeal Membrane Oxygenation for 2009 Influenza A(H1N1) Acute Respiratory Distress Syndrome. JAMA. 2009;302(17):1888–95. 10.1001/jama.2009.1535. 10.1001/jama.2009.1535 19822628

[10] Noah MA, Peek GJ, Finney SJ (2011) Referral to an extracorporeal membrane oxygenation center and mortality among patients with severe 2009 influenza A(H1N1). JAMA 306:1659–1668 21976615 10.1001/jama.2011.1471

[11] Alshahrani MS, Sindi A, Alshamsi F (2018) Extracorporeal membrane oxygenation for severe Middle East respiratory syndrome coronavirus. Ann Intensive Care. 8(1):3. 10.1186/s13613-017-0350-x 29330690 10.1186/s13613-017-0350-x PMC5768582

[12] Henry BM (2020) COVID-19, ECMO, and lymphopenia: a word of caution. Lancet Respir Med 8(4):e24. 10.1016/S2213-2600(20)30119-3 32178774 10.1016/S2213-2600(20)30119-3 PMC7118650

[13] Ma X, Liang M, Ding M, Liu W, Ma H, Zhou X, Ren H. Extracorporeal Membrane Oxygenation (ECMO) in Critically Ill Patients with Coronavirus Disease 2019 (COVID-19) Pneumonia and Acute Respiratory Distress Syndrome (ARDS). Med Sci Monit. 2020;26:e925364. 10.12659/MSM.925364. 10.12659/MSM.925364 PMC7430351 32759887

[14] Stang A (2010) Critical evaluation of the Newcastle-Ottawa scale for the assessment of the quality of nonrandomized studies in meta-analyses. Eur J Epidemiol 25(9):603–605. 10.1007/s10654-010-9491-z 20652370 10.1007/s10654-010-9491-z

[15] DerSimonian R, Laird N (1986) Meta-analysis in clinical trials. Control Clin Trials 7(3):177–188. 10.1016/0197-2456(86)90046-2 3802833 10.1016/0197-2456(86)90046-2

[16] Higgins JP, Thompson SG, Deeks JJ, Altman DG (2003) Measuring inconsistency in meta-analyses. BMJ. 327(7414):557–560. 10.1136/bmj.327.7414.557 12958120 10.1136/bmj.327.7414.557 PMC192859

[17] Zhou, Ning and Wang, Luyun and Jiang, Jiangang and Cheng, Peng and Cui, Guanglin and Wang, Feng and Guan, Zhimin and Zhang, Panpan and Li, Shengqing and Wen Wang, Dao, Application of ECMO successfully treated critically ill COVID-19 patients (4/1/2020). Available at SSRN: https://ssrn.com/abstract=3569889

[18] Zhang G, Hu C, Luo L, Fang F, Chen YF, Li JG, Peng ZY, Pan H (2020) Clinical features and treatment of 221 patients with COVID-19 in Wuhan, China. SSRN Electron J. 10.2139/ssrn.3546095 10.1016/j.jcv.2020.104364 PMC7194884 32311650

[19] Takeda S (2020) Nationwide system to centralize decisions around ECMO use for severe COVID-19 pneumonia in Japan. Acute Med Surg 4:20–21 10.1002/ams2.510 PMC7231563 32431849

[20] Jacobs JP, Stammers AH, St Louis J et al (2020) Extracorporeal membrane oxygenation in the treatment of severe pulmonary and cardiac compromise in COVID-19: experience with 32 patients. ASAIO J 66(7):722–730. 10.1097/MAT.0000000000001185 32317557 10.1097/MAT.0000000000001185 PMC7217117

[21] Yang X, Yu Y, Xu J et al (2020) Clinical course and outcomes of critically ill patients with SARS-CoV-2 pneumonia in Wuhan, China: a single-centered, retrospective, observational study. Lancet Respir Med 2600:1–7 10.1016/S2213-2600(20)30079-5 PMC7102538 32105632

[22] Ruan Q, Yang K, Wang W, Jiang L, Song J (2020) Correction to: Clinical predictors of mortality due to COVID-19 based on an analysis of data of 150 patients from Wuhan, China (Intensive Care Medicine, (2020), 10.1007/s00134-020-05991-x). Intensive Care Med. 10.1007/s00134-020-06028-z 10.1007/s00134-020-06028-z PMC7131986 32253449

[23] Li X, Guo Z, Li B, Zhang X, Tian R, Wu W, Zhang Z, Lu Y, Chen N, Clifford SP, Huang J. Extracorporeal Membrane Oxygenation for Coronavirus Disease 2019 in Shanghai, China. ASAIO J. 2020;66(5):475–81. 10.1097/MAT.0000000000001172. 10.1097/MAT.0000000000001172 PMC7273861 32243266

[24] Guan W, Ni Z, Hu Y, Liang WH, Ou CQ, He JX, Liu L, Shan H, Lei CL, Hui DSC, du B, Li LJ, Zeng G, Yuen KY, Chen RC, Tang CL, Wang T, Chen PY, Xiang J, Li SY, Wang JL, Liang ZJ, Peng YX, Wei L, Liu Y, Hu YH, Peng P, Wang JM, Liu JY, Chen Z, Li G, Zheng ZJ, Qiu SQ, Luo J, Ye CJ, Zhu SY, Zhong NS, China Medical Treatment Expert Group for Covid-19 (2020) Clinical characteristics of coronavirus disease 2019 in China. N Engl J Med 382(18):1708–1720. 10.1056/NEJMoa2002032 32109013 10.1056/NEJMoa2002032 PMC7092819

[25] Zeng Y, Cai Z, Xianyu Y, Yang BX, Song T, Yan Q (2020) Prognosis when using extracorporeal membrane oxygenation (ECMO) for critically ill COVID-19 patients in China: a retrospective case series. Crit Care 24(1):148. 10.1186/s13054-020-2840-8 32293518 10.1186/s13054-020-2840-8 PMC7156900

[26] Beyls C, Huette P, Abou-Arab O, Berna P, Mahjoub Y. Extracorporeal membrane oxygenation for COVID-19-associated severe acute respiratory distress syndrome and risk of thrombosis. Br J Anaesth. 2020;125(2):e260-e262. 10.1016/j.bja.2020.04.079. 10.1016/j.bja.2020.04.079 PMC7198213 32414510

[27] Osho AA, Moonsamy P, Hibbert KA, Shelton KT, Trahanas JM, Attia RQ, Bloom JP, Onwugbufor MT, D'Alessandro DA, Villavicencio MA, Sundt TM, Crowley JC, Raz Y, Funamoto M. Veno-venous Extracorporeal Membrane Oxygenation for Respiratory Failure in COVID-19 Patients: Early Experience From a Major Academic Medical Center in North America. Ann Surg. 2020;272(2):e75-e78. 10.1097/SLA.0000000000004084. 10.1097/SLA.0000000000004084 PMC7373471 32675503

[28] Haye G, Fourdrain A, Abou-Arab O, Berna P, Mahjoub Y (2020) COVID-19 outbreak in France: setup and activities of a mobile Extra Corporeal Membrane Oxygenation (ECMO) team during the first 3 weeks. J Cardiothorac Vasc Anesth 000:8–10 10.1053/j.jvca.2020.05.004 PMC7207137 32457006

[29] Ñamendys-Silva SA (2020) ECMO for ARDS due to COVID-19. Heart Lung 26:S0147–S9563. 10.1016/j.hrtlng.2020.03.012 (20)30100-X Epub ahead of print 10.1016/j.hrtlng.2020.03.012 PMC7194678 32223988

[30] Henry BM, Lippi G (2020) Poor survival with extracorporeal membrane oxygenation in acute respiratory distress syndrome (ARDS) due to coronavirus disease 2019 (COVID-19): pooled analysis of early reports. J Crit Care. 58:27–28. 10.1016/j.jcrc.2020.03.011 32279018 10.1016/j.jcrc.2020.03.011 PMC7118619

[31] Barbaro RP, Odetola FO, Kidwell KM, Paden ML, Bartlett RH, Davis MM, Annich GM (2015) Association of hospital-level volume of extracorporeal membrane oxygenation cases and mortality: analysis of the Extracorporeal Life Support Organization Registry. Am J Respir Crit Care Med 191(8):894–901. 10.1164/rccm.201409-1634OC 25695688 10.1164/rccm.201409-1634OC PMC4435456

[32] ARDS Definition Task Force, Ranieri VM, Rubenfeld GD, Thompson BT, Ferguson ND, Caldwell E, Fan E, Camporota L, Slutsky AS. Acute respiratory distress syndrome: the Berlin Definition. JAMA. 2012;307(23):2526–33. 10.1001/jama.2012.5669. 10.1001/jama.2012.5669 22797452

[33] Gattinoni L, Chiumello D, Rossi S (2020) COVID-19 pneumonia: ARDS or not? Crit Care 24(1):154. 10.1186/s13054-020-02880-z 32299472 10.1186/s13054-020-02880-z PMC7160817

[34] Yin S, Huang M, Li D, Tang N. Difference of coagulation features between severe pneumonia induced by SARS-CoV2 and non-SARS-CoV2. J Thromb Thrombolysis. 2020:1–4. 10.1007/s11239-020-02105-8. 10.1007/s11239-020-02105-8 PMC7124128 32246317

[35] Fina D, Matteucci M, Jiritano F, Meani P, Lo Coco V, Kowalewski M, Maessen J, Guazzi M, Ballotta A, Ranucci M, Lorusso R. Extracorporeal membrane oxygenation without therapeutic anticoagulation in adults: A systematic review of the current literature. Int J Artif Organs. 2020;43(9):570–8. 10.1177/0391398820904372. 10.1177/0391398820904372 32037946

[36] ELSO COVID-19 interim guidelines. ELSO, 2020 (Available from www.elso.org). Accessed 2 June 2020.

[37] Munshi L, Walkey A, Goligher E, Pham T, Uleryk EM, Fan E (2019) Venovenous extracorporeal membrane oxygenation for acute respiratory distress syndrome: a systematic review and meta-analysis. Lancet Respir Med. 7(2):163–172. 10.1016/S2213-2600(18)30452-1 30642776 10.1016/S2213-2600(18)30452-1

[38] Mehta P, McAuley DF, Brown M, Sanchez E, Tattersall RS, Manson JJ, HLH Across Specialty Collaboration, UK (2020) COVID-19: consider cytokine storm syndromes and immunosuppression. Lancet 395(10229):1033–1034 32192578 10.1016/S0140-6736(20)30628-0 PMC7270045

[39] Abrams D, Ferguson ND, Brochard L, Fan E, Mercat A, Combes A, Pellegrino V, Schmidt M, Slutsky AS, Brodie D (2019) ECMO for ARDS: from salvage to standard of care? Lancet Respir Med. 7(2):108–110. 10.1016/S2213-2600(18)30506-X 30642778 10.1016/S2213-2600(18)30506-X

